# Novel nonlinear reconstruction method with grey-level quantisation units for electron tomography

**DOI:** 10.1038/s41598-020-77156-1

**Published:** 2020-11-19

**Authors:** Norio Baba, Kenji Kaneko, Misuzu Baba

**Affiliations:** 1 Major of Informatics, Graduate School, Kogakuin University, 2665-1 Nakano, Hachioji, Tokyo 192-0015 Japan; 2 Department of Materials Science and Engineering, Kyushu University, Fukuoka, 819-0395 Japan; 3 Research Institute for Science and Technology, Kogakuin University, 2665-1 Nakano, Hachioji, Tokyo 192-0015 Japan

**Keywords:** Engineering, Nanoscience and technology

## Abstract

We report a new computed tomography reconstruction method, named quantisation units reconstruction technique (QURT), applicable to electron and other fields of tomography. Conventional electron tomography methods such as filtered back projection, weighted back projection, simultaneous iterative reconstructed technique, etc. suffer from the ‘missing wedge’ problem due to the limited tilt-angle range. QURT demonstrates improvements to solve this problem by recovering a structural image blurred due to the missing wedge and substantially reconstructs the structure even if the number of projection images is small. QURT reconstructs a cross-section image by arranging grey-level quantisation units (QU pieces) in three-dimensional image space via unique discrete processing. Its viability is confirmed by model simulations and experimental results. An important difference from recently developed methods such as discrete algebraic reconstruction technique (DART), total variation regularisation—DART, and compressed sensing is that prior knowledge of the conditions regarding the specimen or the expected cross-section image is not necessary.

## Introduction

Electron tomography (ET) has the potential to analyse three-dimensional (3D) structures of nanomaterials^1^ and the 3D morphology of biological samples^2,3^. Although ET is common in diverse fields, the ‘missing wedge’ problem due to the limited tilt-angle range remains a serious issue. This problem hinders the interpretation of a structure appearing in a cross-section image because it distorts the structural image and blurs the cross-section image.

Many proposals have been reported to address this issue, including those that are not directly for ET purposes. A unique mathematical operator named the lambda reconstruction operator demonstrates that streak artefacts caused by the Fourier integral calculus, which is due to the limited tilt-angle range, is mitigated in micro local analysis^4,5^. An interesting method is an image denoising method based on a statistical approach^6^. Briefly, white Gaussian noise is added to the 3D input tomogram volume, and the original 3D Fourier coefficients before the noise addition are substituted in the 3D spectrum after the noise addition. Next, a 3D denoising algorithm, basically BM4D (Block Matching 4D) algorithm^7,8^, is executed. Due to its random nature, a smaller amount of added noise appears close to the signal. Consequently, the signal is preserved after denoising. This procedure is iteratively performed by adding an evaluation step based on a dedicated Markov Chain Monte Carlo framework. This article^6^ describes that the method makes the best statistical guess of what the missing data could be based on what has been observed. As a result, spectral recovery in the missing wedge domain is verified.

Recently, sparse modelling and regularisation methods have been applied to various restoration and inverse problems. For ET to solve this issue, methods of the discrete algebraic reconstruction technique (DART)^9,10^, total variation regularisation (TVR)—DART^11^, and compressed sensing (CS)^12–15^ have been proposed. Under the sparse condition given in each method based on prior knowledge regarding the specimen or the expected cross-section image, artefacts due to the issue are reduced. These methods also decrease the required number of projection images. However, even with the aforementioned advances, the missing wedge problem has yet to be fully resolved.

To overcome this deficiency, machine learning methods for image restoration have recently been proposed^16,17^. These methods require a very large collection of image patch pairs between artificial and artefact-free ones for a learned dictionary. On the other hand, this study aims to improve the reconstruction method itself.

Our research strives to create a unique reconstruction method to fully address this problem^18,19^. In the early stage, single component objects were the targets to be reconstructed. They were a Pt super crystal^18^ and Pt/C replica specimens of biological samples^19^. In principle, the projection image intensity distribution of such an object is proportional to the thickness distribution of the object along the tilt direction. A unique reconstruction method was devised by modifying the simultaneous iterative reconstructed technique (SIRT) algorithm to reconstruct a binary cross-section image. This image then becomes the rough object-occupied region when the process converges. A final real cross-section image is obtained with a regular SIRT, where the image reconstruction is limited inside the region. As a result, the missing wedge artefact is considerably reduced because the region to be reconstructed is limited. The way of this thinking has been extended toward the development of the present method.

Our method in this paper does not require prior knowledge or conditions regarding the specimen or the expected cross-section image. It only needs tilt series images and tilt angle data. From the user’s viewpoint, the proposed method can be categorised as a method similar to filtered back projection (FBP)^20,21^, weighted back projection (WBP)^20^, SIRT (e.g.,^22^), etc. Nevertheless, our method provides powerful effects. It can recover a structural image blurred by a missing wedge and reconstruct the structure even if the number of projection images is small.

**Figure 1:**
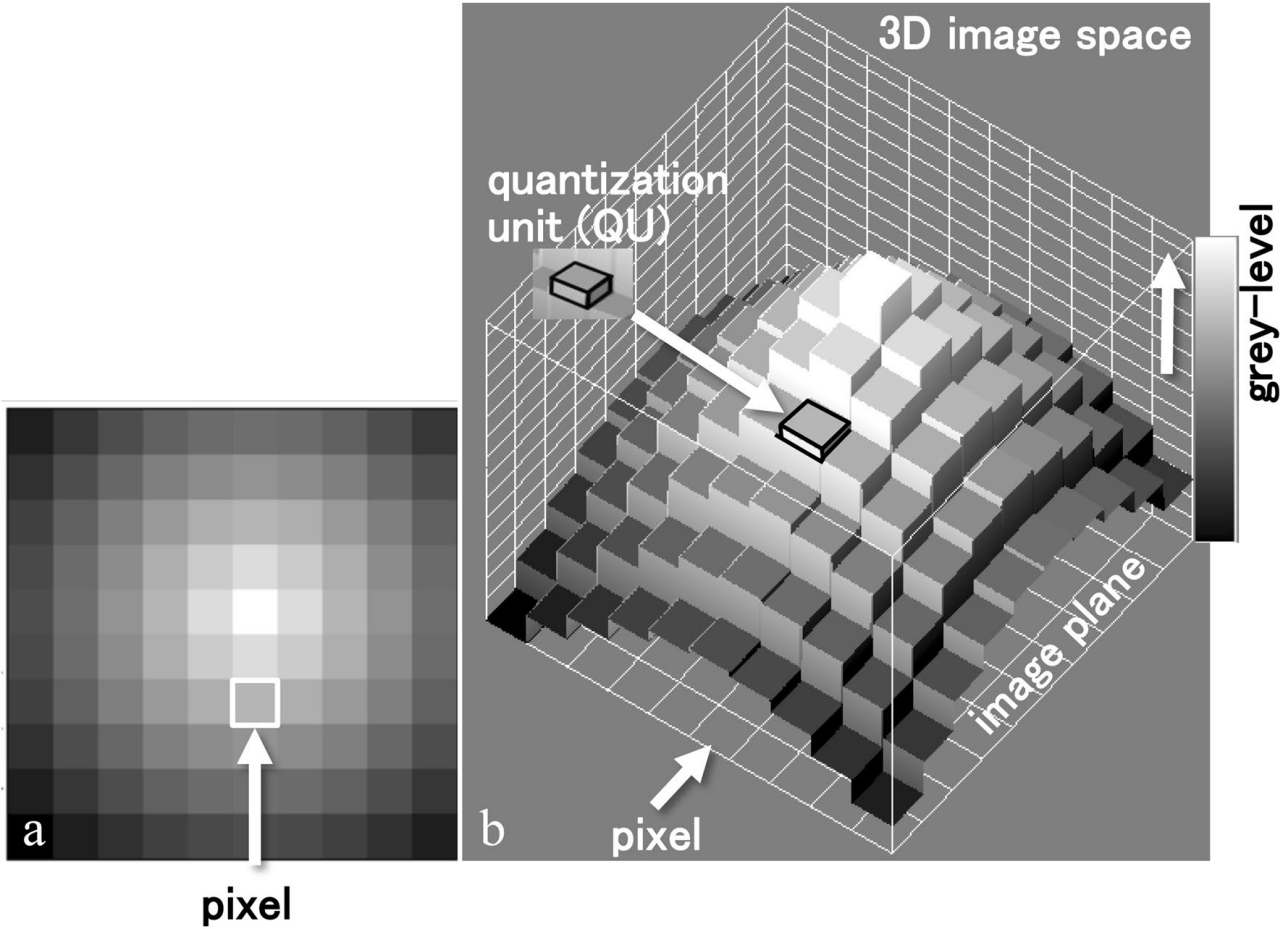
3D representation of an image using quantisation units (QUs). (**a**) Enlargement to show pixels. (**b**) 3D image space representing the sample image of (**a**). Present method reconstructs a cross-section image by stacking QU pieces.

In the proposed method, the interpretation of an image itself is redefined. A digital image is created by a quantisation process of an analogue image. Then the grey level in a pixel due to quantisation can be defined as the number of quantisation units (QUs). As shown in Fig. 1 the digital image is represented in a 3D space whose axes are two-dimensional (2D) image coordinates and the number of QUs. Therefore, the image can be made by stacking a finite number of QU pieces. This finite number equals a numerical value for the integration of a cross-section image.

**Figure 2:**
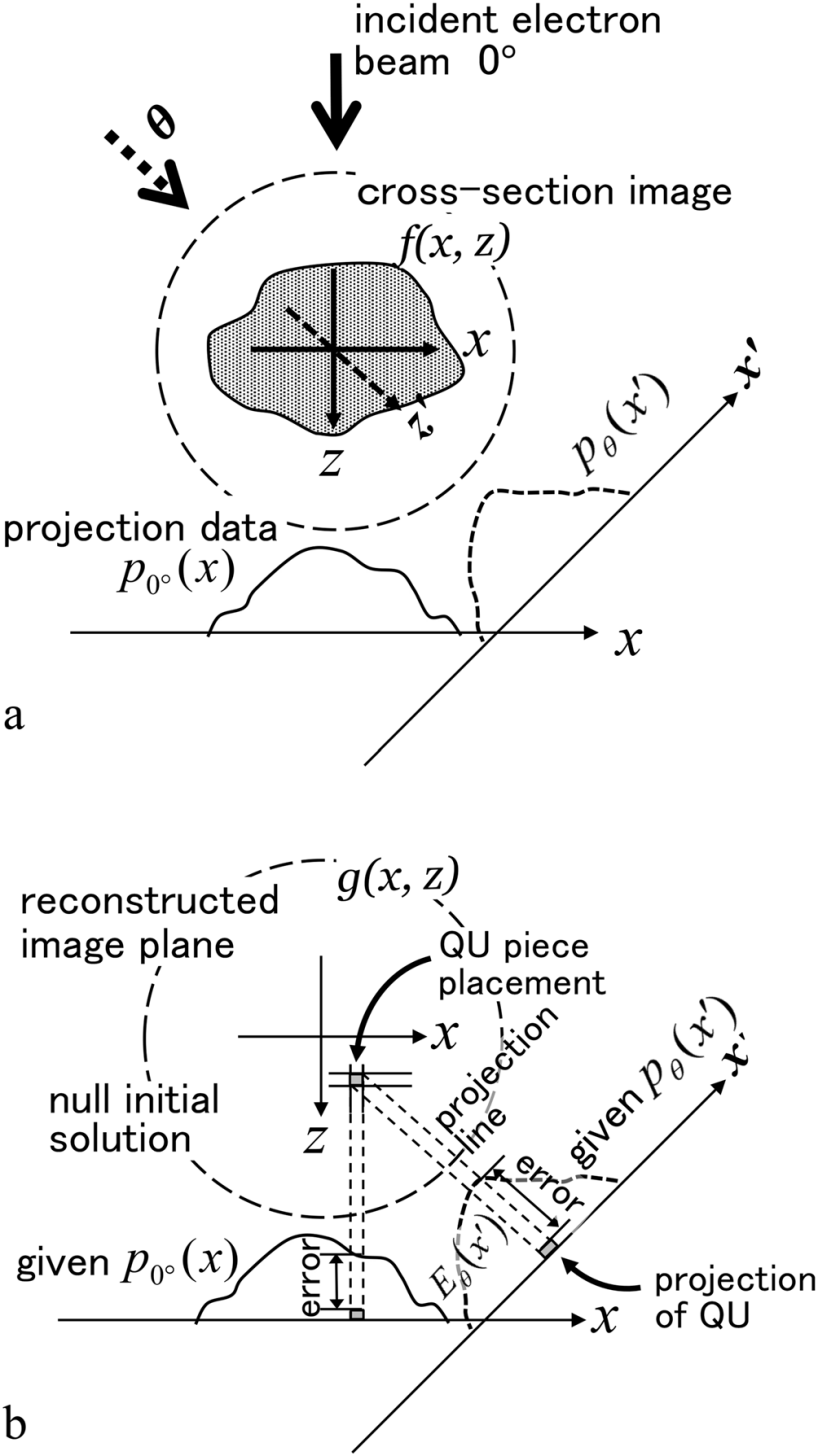
Schematic representation for the concept of the error map $E_{map} \left( {x,z} \right)$. (**a**) Relationship between a cross-section image and projection data. (**b**) Residual errors between the projection data by placing a QU piece with a certain value (q) and the given projection data for reconstruction. Cross-section image is reconstructed by arranging the QU pieces. As a basic explanation, a QU piece is put in an arbitrary pixel position in the initial reconstruction stage (null solution). Then arrangement of QUs is performed to reduce the errors. QU placement position depends on the value calculated by summing up the errors with respect to all projection data before the QU placement. In this text, a map of such values in all pixels is called as the error map $E_{map} \left( {x,z} \right)$. In principle, the maximum value position should be selected as the QU placement in each reconstruction step. Due to the nonlinear change in the error map upon QU piece placement, the value ‘q’ of QU is not a simple relation.

As shown in Fig. 2a, the number of QU pieces can be calculated by a numerical integration of the projection data [one-dimensional (1D) as a function of the ‘x’ coordinate] independent of the tilt direction. In addition, the projection data is the distribution of the number of QU pieces in each x belonging to the ray line perpendicular to x within the cross-section image. Under these constraints for the number of QU pieces, we devise a new reconstruction method, called QURT (Quantisation Units Reconstruction Technique), where innumerable QU pieces are sophisticatedly arranged in the 3D image space of the cross-section plane. The above limited number of QU pieces can successfully reconstruct the cross-section image and is unaffected by the missing wedge. This constraint condition and the novel manner using the QU pieces is applicable even if the number of projection images is small.

## Results

### Constraint condition and the grey-level QU

A basic strategy to overcome the missing wedge problem is to determine useful prior knowledge^23^ and devise a calculation algorithm that satisfies the knowledge. In our method (QURT), the integrated value of the projection data (1D distribution) is effective knowledge as it is equal to the 2D integrated value of a cross-section image (Fig. 2a). This relationship is accurate for any projection direction in the ideal case. That is, the object is projected inside the imaging plane without missing parts in the tilt direction.

This relationship can be understood as follows. In linear projection theory, the projection data $p_{\theta } \left( {x^{{\prime }} } \right)$ of the cross-section image $f\left( {x,z} \right)$ is obtained by integrating with $f\left( {x,z} \right)$ along the line perpendicular to the x’ axis, where the line is parallel to the tilt direction θ. Therefore, the 2D integration of $f\left( {x,z} \right)$ is equal to the 1D integration of $p_{\theta } \left( {x^{{\prime }} } \right)$ along x′, and the integrated value is a constant, which is independent of the tilt angle as (Fig. 2a)

1 $$\int {p_{0^\circ } \left( x \right)\;dx} = \int {p_{\theta } \left( {x^{{\prime }} } \right)\;dx^{{\prime }} = } \iint {f\left( {x,z} \right)\,dxdz} = const.$$

To adapt the constraint condition in Eq. (1), we devised a novel method to reconstruct a cross-section image. The constant value obtained by Eq. (1) is approximately given as an integer number ‘N’. Here, it is recognised that a digital image is made by quantisation. If a grey-level QU is defined as ‘q’ with a small enough value, a pixel grey value is the number of stacked units (QU pieces). Then the total number of QU pieces needed to generate the cross-section image is approximated to N as

2 $$N \cdot q = q\sum\limits_{j} {n_{\theta } \left( {x_{j}^{{\prime }} } \right)} \approx \sum\limits_{j} {p_{\theta } \left( {x_{j}^{{\prime }} } \right)} = \sum\limits_{j} {\sum\limits_{k} {f\left( {x_{j}^{{\prime }} ,z_{k}^{{\prime }} } \right)} }$$

where $n_{\theta } \left( {x_{j}^{{\prime }} } \right)$ is the distribution of the number of QU pieces in each $x_{j}^{{\prime }}$. j and k are index numbers for discrete coordinates x′ and z′, respectively. z′ is the coordinate parallel to the tilt direction θ.

### Reconstruction with grey-level QUs

Here we describe our reconstruction method (QURT) using the finite number N of QU pieces. QURT correctly arranges all QU pieces in the cross-section image plane (x, z) by stacking along the grey-level direction while minimising errors of the projection data for all tilt angles.

First, a certain tilt angle is selected as a base angle ‘Θ’ for the reconstruction. The total number N of QU pieces (integer number) is determined as

3 $$N \approx {{\left( {\sum\limits_{j} {p_{\Theta } \left( {x_{j}^{{\prime }} } \right)} } \right)} \mathord{\left/ {\vphantom {{\left( {\sum\limits_{j} {p_{\Theta } \left( {x_{j}^{{\prime }} } \right)} } \right)} q}} \right. \kern-\nulldelimiterspace} q},\;{\text{and}}\;n_{\Theta } \left( {x_{j}^{{\prime }} } \right) \approx {{p_{\Theta } \left( {x_{j}^{{\prime }} } \right)} \mathord{\left/ {\vphantom {{p_{\Theta } \left( {x_{j}^{{\prime }} } \right)} q}} \right. \kern-\nulldelimiterspace} q}.$$

Thus, the number of QU pieces is determined in each $x_{j}^{{\prime }}$ column. This is the constraint condition.

The next step is determining how to place the finite number of QU pieces in the cross-section image plane (x′, z′). The basic idea is derived from a study regarding the error between a single QU piece put in a (x′, z′) pixel position and all given projection data (see Fig. 2b). The QU piece must be placed in a specific position to minimise the error. A certain error amount is added when the projection data for all tilt directions changes by placing a QU piece in a (x′, z′) position. If the above-mentioned error is the total value summing up the differences to subtract the given projection data from the current calculated projection data for all tilt directions, a QU piece should be placed in the largest negative error position because the error in the (x′, z′) position indicates the degree of a lack of the QU piece. If the error value is the plus amount, the QU in that position should be erased. This error is calculated as a map of $E_{map} \left( {x,z} \right)$ in the cross-section image plane with a simple back projection of all such differences in the projection data described above. Finally, when the correct cross-section image (correct arrangement of QU pieces) is reconstructed, the $E_{map} \left( {x,z} \right)$ becomes a zero distribution. Therefore, determining the method to place the finite number of QU pieces to let $E_{map} \left( {x,z} \right)$ become zero is important. $E_{map} \left( {x,z} \right)$ is given as

4 $$\begin{aligned} & E_{map} \left( {x,z} \right) = BP\left[ {E_{{\theta_{i} }} \left( {x^{{\prime }} } \right)} \right] \\ & E_{{\theta_{i} }} \left( {x^{{\prime }} } \right) = p_{{\theta_{i} {\text{ cal}}{.}}} \left( {x^{{\prime }} } \right) - p_{{\theta_{i} \,\exp .}} \left( {x^{{\prime }} } \right) \\ & p_{{\theta_{i} \,{\text{cal}}{.}}} \left( {x^{{\prime }} } \right) = FP\left[ {g\left( {x,z} \right)} \right] \\ \end{aligned}$$

where $BP[]$ is a simple back-projection, which is equal to FBP without filtering. $E_{{\theta_{i} }} \left( {x^{{\prime }} } \right)$ is the error distribution to subtract the given projection data $p_{{\theta_{i} {\text{ exp}}{.}}} \left( {x^{{\prime }} } \right)$ from the current calculated projection data $p_{{\theta_{i} \;{\text{cal}}{.}}} \left( {x^{{\prime }} } \right)$. $FP[]$ is a projection calculation in the θ_i_ direction. $g\left( {x,z} \right)$ is the current reconstructed image in the iterative procedure. Altering the QU piece arrangement changes $E_{map} \left( {x,z} \right)$ nonlinearly. Due to the complicated arrangement even if the change is limited to one pixel position only, the simple back projection alters the map of the whole area of the cross-section image plane.

**Figure 3:**
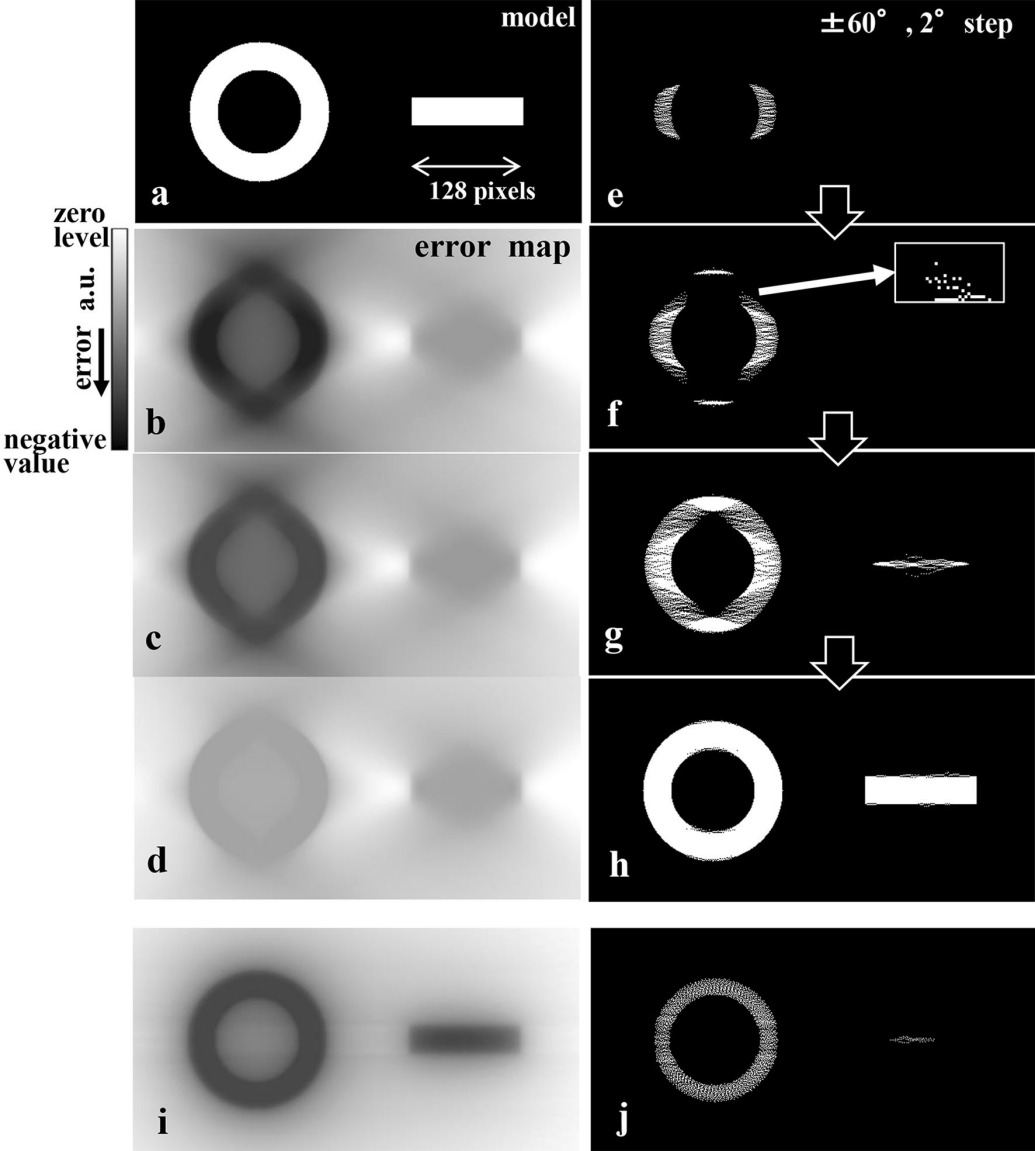
Examination with a simple binary model using the basic reconstruction procedure. (**a**) Model susceptible to the missing wedge. (**b**–**d**) Changes in the error maps from the initial stage (**b**), halfway (**c**), and final reconstruction (**d**) for a test condition of ± 60° with 2° steps. Error maps are affected by the missing wedge due to the calculation with a simple back projection under the limited angular range condition. (**e**–**h**) Process of the present reconstruction using QUs (small white dots shown in the enlarged image inserted in (**f**)) from the initial stage (**e**) via (**f**) and (**g**) to the final reconstruction (**h**). QUs are arranged one-by-one in the negative maximum error position in the current error map, which is updated after each QU placement. In the initial stage, QUs are not arranged in the affected parts of the error map because the error amount in these parts is relatively small compared to those in the structural parts as shown in (**e**). Later, QUs are not arranged in the affected parts because the error amount in both parts gradually becomes smaller. Eventually a finite number of QUs are placed, and the missing wedge artefact in the cross-section image is avoided. (**i** and **j**) Error map in a near initial stage and the corresponding halfway QU arrangement for the almost ideal condition (tilt-angle range ± 90° with 2° steps). Although the error map is no longer distorted, QUs are not uniformly arranged because the error amount differs among structural parts.

Before providing the entire explanation, we describe the basic procedure to place the QU pieces. In the procedure, they are placed one-by-one in the negative maximum error position in the current error map of $E_{map} \left( {x,z} \right)$. The error map is updated after each QU placement. This process is repeated until the number of QU pieces reaches the limit of N under the second equation in Eq. (3) (Fig. 3).

Because the basic procedure is only applicable to a very simple binary model (Fig. 3a), we improved the procedure so that it can accommodate general specimens. All procedures have the same purpose, which is to converge into the correct cross-section image. In other words, all are measures that avoid a local solution trap (e.g., the local minimum error) in the nonlinear optimisation. The major improved procedure changes the size and the value of the QU piece in the iterative reconstruction.

**Figure 4:**
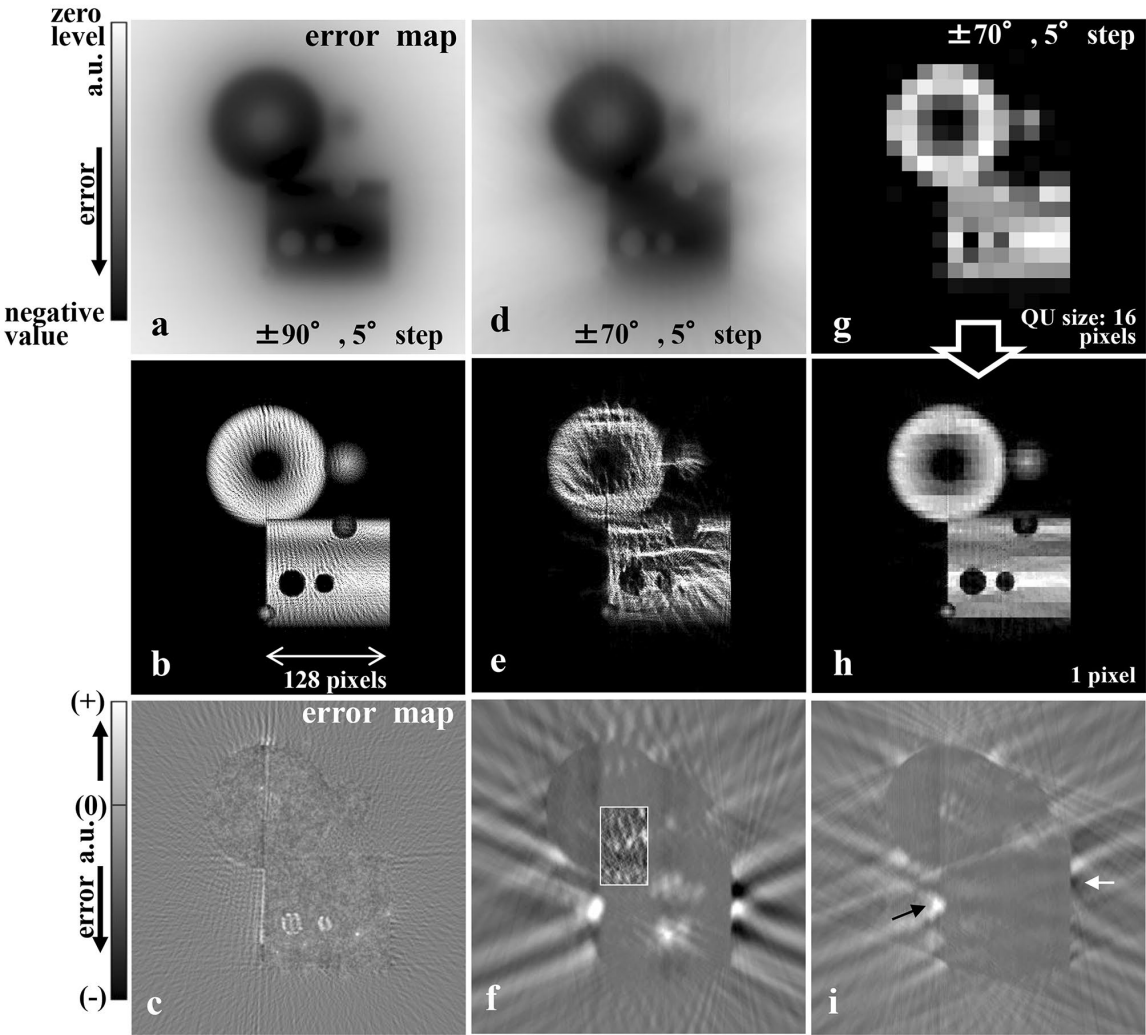
Examinations with the general model shown in Fig. 5a using the basic reconstruction procedure and the improved procedure with a single base tilt angle (Θ). (**a**–**c**) Results for the nearly ideal conditions (tilt-angle range ± 90° with 5° steps) using the basic procedure. (**a**) Initial error map. (**b**) Reconstructed result of an artificial granular image created with many QU dots. (**c**) Final error map showing a granular distribution due to a discrete fine QU dots arrangement. Even with the full tilt-angle range, the basic procedure fails in reconstruction of the general model. (**d**–**f**) Results for the missing wedge case (tilt-angle range ± 70° with 5° steps) using the basic procedure. (**d**) Initial error map. (**e**) Similar reconstruction of an artificial wave-like granular image. (**f**) Final error map of the remaining errors, especially outside the model structure with a granular distribution inside the structure due to discrete fine QU dots (see centre inset of the amplified partial map). For a limited tilt-angle range, the result (**e**) is drastically degraded compared to (**b**). (**g**–**i**) Results for the same missing wedge case using the improved procedures with a single base tilt angle (Θ = 0°) is used. (**g**) Reconstruction in the image definition of $32 \times 32$ pixels (QU piece size is 16 pixels). (**h**) Final reconstruction is clearly improved compared to (**e**), although some mosaic-like patterns are shown. (**i**) Final error map contains errors (see arrows) due to the incomplete mosaic-like patterns. Improved procedures to change the size of QU piece, large (32 pixels square as a standard case) to fine (1 pixel), in an iterative reconstruction manner and refinement of the current arrangement of QU pieces moving inside a specific $x^{\prime}_{j}$ column are effective (See text for details).

The first improvement has the following steps. First assign the QU piece to a large size (e.g., 32 pixels square). Second, reduce the size in a stepwise manner until it reaches a one-pixel size. Once the size is unchanged at the one-pixel level, the cross-section image becomes an artificial granular image composed of many QU dots (Fig. 4b,e). The process of placing a one-pixel size QU piece induces a change in $E_{map} \left( {x,z} \right)$ with one-pixel fineness. In a nonlinear optimisation, this often falls into a local minimum because the manner is too fine.

Similar to the above procedure for the size of the QU piece, the value (q) of QU is roughly changed stepwise until it reaches the final smallest value. In practice, the final smallest value of q is set to one. According to the adjustment need of the image intensity, the value of the projection data is amplified. Note that the change in the q value helps reduce the calculation time. Even if the value is kept at one during the iterative reconstruction, the cross-section image is correctly reconstructed. However, a long calculation time is necessary.

In the second improvement, the QU pieces are rearranged to further reduce the error after the number of QU reaches the defined N. This rearrangement is realised as QU piece movements. Each movement is performed inside a certain $x_{j}^{{\prime }}$ column while maintaining the condition of $n_{\Theta } \left( {x_{j}^{{\prime }} } \right)$. The movement is iteratively repeated by moving the one with the largest reducible error based on the current $E_{map} \left( {x,z} \right)$. As shown in Fig. 4g,h of the model simulation, the first and the second improved procedures produce the desired effect, but the error map does not sufficiently converge into the flat zero distribution (see Fig. 4i, arrows). Instead, mosaic-like images are observed in the final reconstructed image (Fig. 4h).

**Figure 5:**
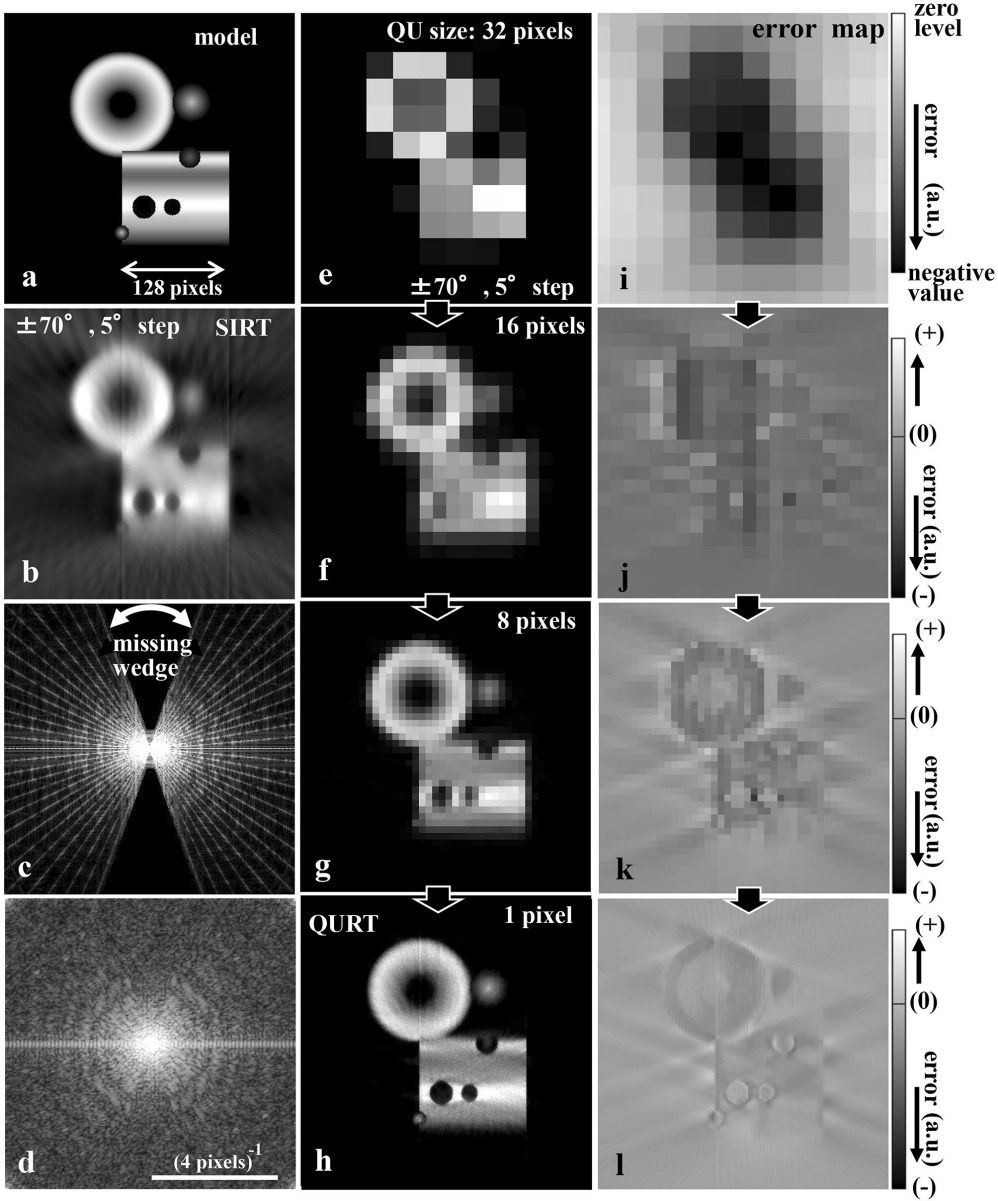
Reconstruction process with the present method (QURT) using all improved reconstruction procedures. (**a**) General model susceptible to the missing wedge used in the simulation. (**b**) Reconstructed result with the conventional SIRT from ± 70°, 5° step projection data. Missing wedge causes considerable blur. (**c**) Fourier transform pattern of (**b**) showing a ‘missing wedge’ and radial spectral lines based on the projection slice theorem. (**d**) Fourier transform pattern of the present reconstruction (QURT) result of (**h**), which recovers the missing wedge spectral region and all the gaps among those radial lines. Typical ring-like diffraction pattern generated from the doughnut model is seen. (**e**–**h**) Convergence process from low to high image definition (16 × 16, 32 × 32, 64 × 64, and 512 × 512 pixels (final)). In this process, three base tilt angles Θ are utilised − 70°, 0°, and + 70°, effectively improving the recovery of the structure rather than the single Θ case shown in Fig. 4h. Final image (**h**) recovers the model without artificial blur. (**i**–**l**) Error maps corresponding to (**e**)–(**h**), respectively. As the model is gradually recovered, the error map converges to the flat zero distribution.

Thus, a third improvement procedure is introduced, where plural base tilt angles Θ are set to generate a cross-section image solution according to each constraint condition of $n_{\Theta } \left( {x_{j}^{{\prime }} } \right)$. After the output of each solution, the current solution is updated by averaging their plural ones. Figure 5 shows a typical reconstruction process incorporating all the improved procedures.

### Flow of the procedure

**Figure 6:**
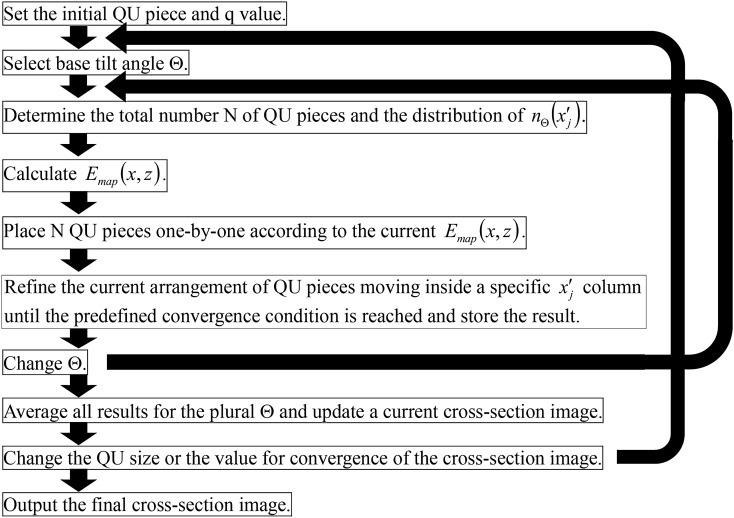
Block flow of the reconstruction procedure. Outer loop changes the size of the QU piece accompanying its value (q), large (32 pixels square as a standard case) to fine (1 pixel). Stepwise change in the q value reduces the calculation time (e.g., a few steps, 8 to 1). However, it is almost irrelevant to the convergence regarding the recovery of the structure. Inner loop changes the plural base tilt angles (Θ) (the minus and the plus maximum tilt angles, and 0° as a standard), which gives the different constraint conditions of $n_{\Theta } \left( {x^{\prime}_{j} } \right)$ in Eq. (3). This is especially effective to remove artificial images (mosaic-like pattern shown in Fig. 4h) due to the square shape of QU pieces in the initial and the halfway size stage. Main part of the processing inside the loops is the calculation of the error map $E_{map} \left( {x,z} \right)$, placement of QUs updating the error map, and refinement of the current arrangement of QUs also updating the error map. QU piece placement and refinement movement depend on the current error map.

Below the reconstruction procedure is described and the procedure is shown in block flow in Fig. 6. (a) Set the initial size of the QU piece (32-pixel square as a standard) and the initial value (q) of QU (typically with an nth power of 2). Subsequently, null is set in the initial cross-section image. (b) Select the base tilt angle Θ. (c) Determine the constraint conditions for the total number N of QU pieces and the distribution of the number of QU as a function of $x^{\prime}_{j}$ column $n_{\Theta } \left( {x_{j}^{{\prime }} } \right)$. (d) Calculate $E_{map} \left( {x,z} \right)$. The map indicates the degree (absence or excess) of the QU piece in every pixel calculated from all the projection data and the current projection data of the most recent cross-section image. (e) Place many QU pieces to the number of N one-by-one in the maximum error pixel in the current error map of $E_{map} \left( {x,z} \right)$, which is updated after each QU placement under the condition of $n_{\Theta } \left( {x_{j}^{{\prime }} } \right)$. (f) Selectively repeat QU piece movements inside the certain $x^{\prime}_{j}$ column while maintaining the condition of $n_{\Theta } \left( {x_{j}^{{\prime }} } \right)$ until a predefined convergence condition, which further reduces the error between the current reconstructed image and all given projection data, is reached. (g) Store the result of (f) into a memory, and repeat steps (b) to (f) while changing Θ to the predetermined other plural base tilt angles. When this repeat is finished, average the results to update a reconstruction result for the current QU piece size and for the current QU value (q). Repeat these reconstruction steps for Θ and average the repeated results for every QU piece size until converging on the final pixel size and q value of one. For example, the size and the value decrease step-by-step as follows: (32 and 8), (32 and 1), (16 and 8), (16 and 1), (in sequence), and the last (1 pixel and the value 1).

### Examine with models

As an underlying examination, the basic reconstruction procedure described above was applied to a binary model (Fig. 3a). This model is made with a simple doughnut and bar. The base tilt angle Θ is 0° (the vertical direction in the model). The test condition is that the tilt angle ranges ± 60° with a step angle of 2°. In process (c) described in the above section, the constraint conditions of the total number N of QU pieces and the distribution of the number of QU as a function of $x^{\prime}_{j}$ column $n_{\Theta } \left( {x_{j}^{{\prime }} } \right)$ are determined from the calculated projection data of the model. Figure 3e–h depict the iterative reconstruction process in the procedure (e) with a 1-pixel size QU piece (value: 1), while Fig. 3b–d show the corresponding $E_{map} \left( {x,z} \right)$, where the white dots denote the QU pieces. Every $E_{map} \left( {x,z} \right)$ in these processes has a negative distribution (black colour), where the depth of black is proportional to the negative value. In each iteration, the QU pieces are placed in high negative regions of the map, and the map gradually changes toward the flat zero distribution. Although $E_{map} \left( {x,z} \right)$ is incomplete and distorted due to the limited tilt-angle range, the model is almost perfectly recovered (Fig. 3h). Figure 3i,j show the near ideal $E_{map} \left( {x,z} \right)$ and an example of a halfway result in the iterative reconstruction for a condition of ± 90° with 2° steps, respectively.

The basic reconstruction procedure fails when applied to general models and experimental samples. Figure 4 shows the result when applying the general model (Fig. 5a) to various structural elements. For the limited tilt-angle range (± 70° with 5° steps), many QU dots create an artificial granular image, which is almost limited to inside the model (Fig. 4e). Even for a case of the full tilt-angle range (± 90° with 5° steps), a similar artificial granular image is reconstructed, but a well-balanced image can be made from the constant dot density (Fig. 4b).

We then applied the improved procedures described in the ‘Flow of the procedure’ section. Figure 5 shows the results of the improved procedure using the same test conditions as those for Fig. 4e. Three base tilt angles Θ are utilised − 70°, 0°, and + 70°, and the initial size of the QU piece is 32 pixels squared. The size is reduced by half stepwise until it reaches 1 pixel. In all processes, the value of QU is kept at 1. The convergence condition is that the change rate of the reconstructed image measured in intervals of 100 iterations is less than 1.0E − 6. Figure 5e–h show the results of the iterative reconstruction progress.

This stepwise reconstruction from rough to fine avoids the local solution trapping problem. In each step, QU pieces are rearranged after the first arrangement of the necessary number QUs specified in the constraint condition. This refinement effectively avoids the problem. Furthermore, by selecting plural base tilt angles of Θ, an average solution can be used to avoid the problem because different plural constraint conditions regarding $n_{\Theta } \left( {x_{j}^{{\prime }} } \right)$ can be set. To clearly highlight this effect, Fig. 4g,i show the result by selecting a single base tilt angle of Θ (0°). Although the halfway reconstructed images until 16 pixels size do not differ significantly from the plural base tilt angles case (Fig. 5f), as the QU piece size is decreased below 16, the level of convergence is relatively worse because some mosaic-like images appear (Fig. 4h).

Finally, these improvements realize the optimum solution (Fig. 5h). Compared to Fig. 4f,i, the corresponding error map (Fig. 5l) converges to the flat zero distribution. Figure 5b shows the corresponding result reconstructed with the conventional method (SIRT). The missing wedge artefact is seen in the Fourier transform pattern (Fig. 5c) of the image. On the other hand, QURT result shows no such artefact (Fig. 5d), which is the Fourier transform of Fig. 5h. Hence, the Fourier spectra are generated in the missing part.

**Figure 7:**
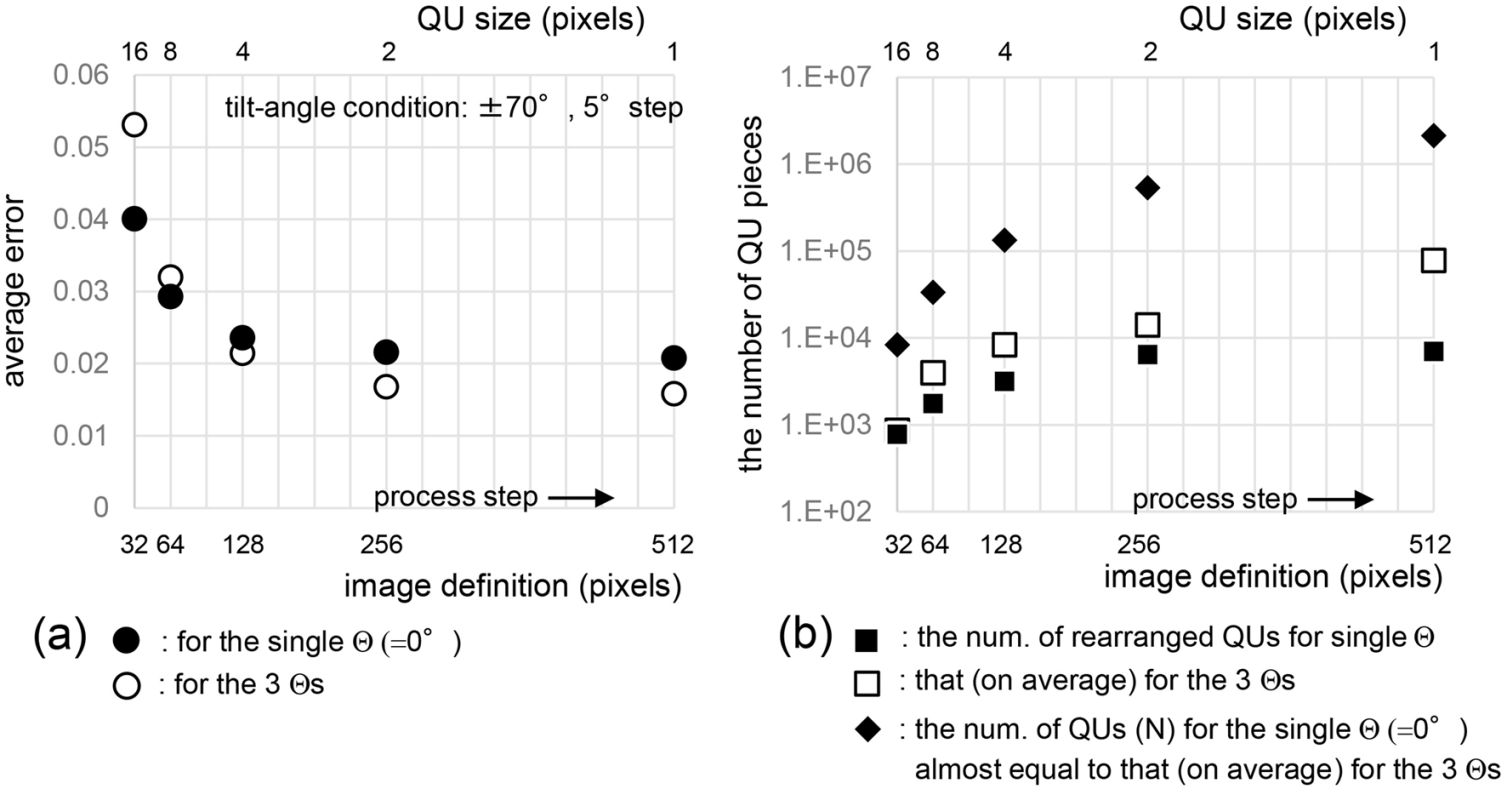
Transition of the average reconstruction error and the number of QU pieces used in each reconstruction process step of the model simulation. (**a**) Each average error calculated between the model and the reconstructed image is normalised by dividing by the maximum value of the model grey-level. Error is gradually reduced as the image definition increases. At a QU size of 4 pixels (128 image definition), further error reduction becomes negligible, and the reconstructed structure roughly appears at this step. (**b**) Number of QUs used in the reconstruction and the number rearranged in each step. There is a large difference for the number of rearranged QUs between the single base tilt angle and the plural ones, suggesting that the effect of a large number of rearranged QUs when using the plural base angles is the removal of the artificial mosaic-like pattern shown in Fig. 4h. However, the difference of the errors for the same choice is small as shown in (**a**).

Figure 7a shows the convergence process from the viewpoint of the transition of the average reconstruction error as a function of the QU piece size, which is related to the image definition and corresponds to Fig. 5f–h. Around a QU size of four pixels, the error is not significantly decreased, and the reconstructed structure roughly appears at this step. This feature is also observed experimentally. Figure 7b shows the transition of the number of QUs (N) needed for the arrangement and the number of rearranged QUs in refinement procedure (f) in the ‘Flow of the procedure’ section. The rate of the increasing number of N between the image definition steps is four. There is a large difference in the number of rearranged QUs between the single base tilt angle and the plural ones. Because the difference in the errors for the same choice is small, as shown in Fig. 7a, the artificial mosaic-like pattern shown in Fig. 4h is removed by rearranging a large number of QUs.

**Figure 8:**
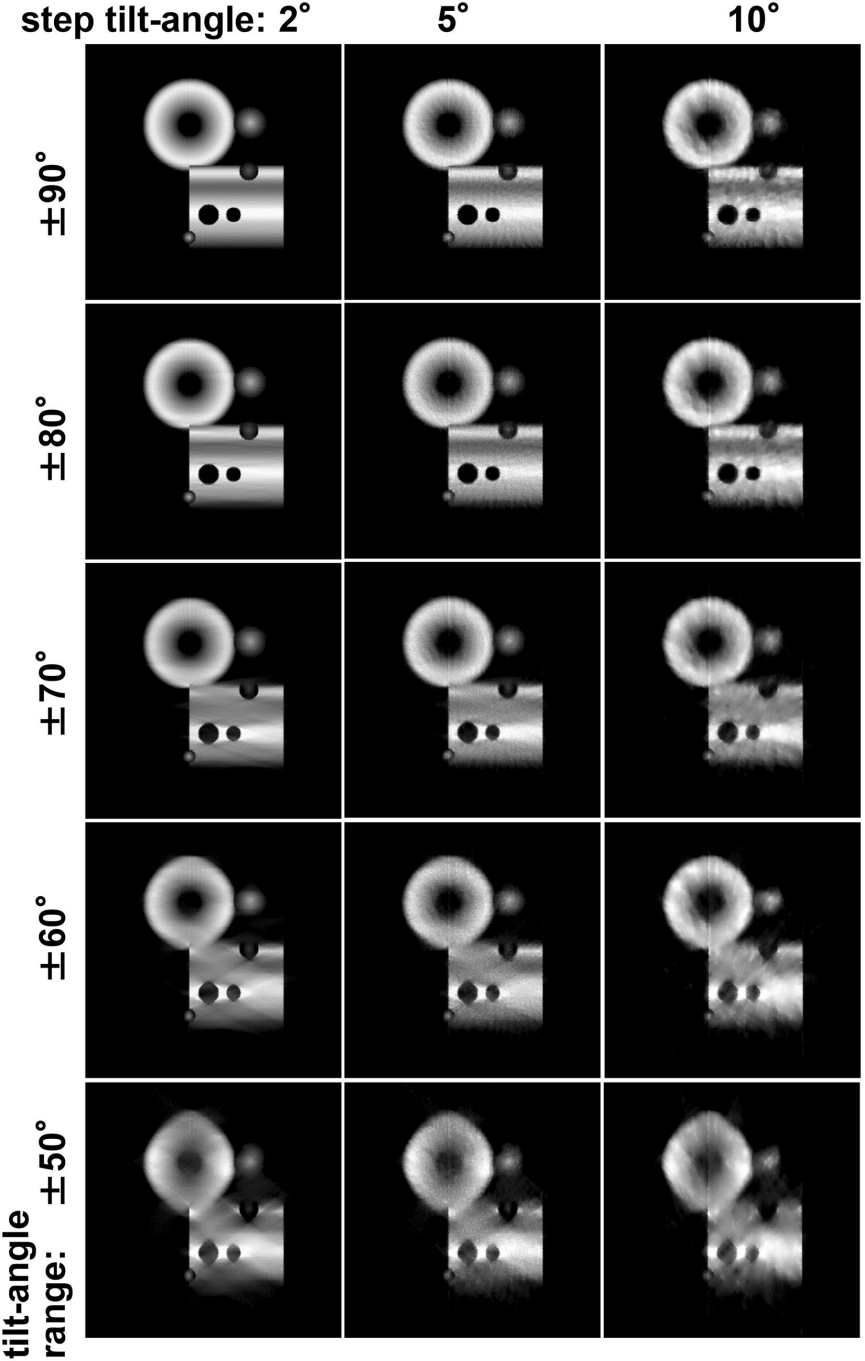
Reconstruction results of the model with QURT for various tilt-angle conditions. Although the missing wedge due to the poorer conditions gradually degrades the image quality from the model (Fig. 5a), the structural parts are well preserved compared to those with the conventional methods shown in Fig. 9. In addition, artificial images such as streaks and blurred background do not appear, and the edge parts are clearly resolved. From a rough judgement, if the tilt angle range is greater than or equal to about ± 60° and the step angle is less than or equal to about 5°, the structure is almost recovered.

**Figure 9:**
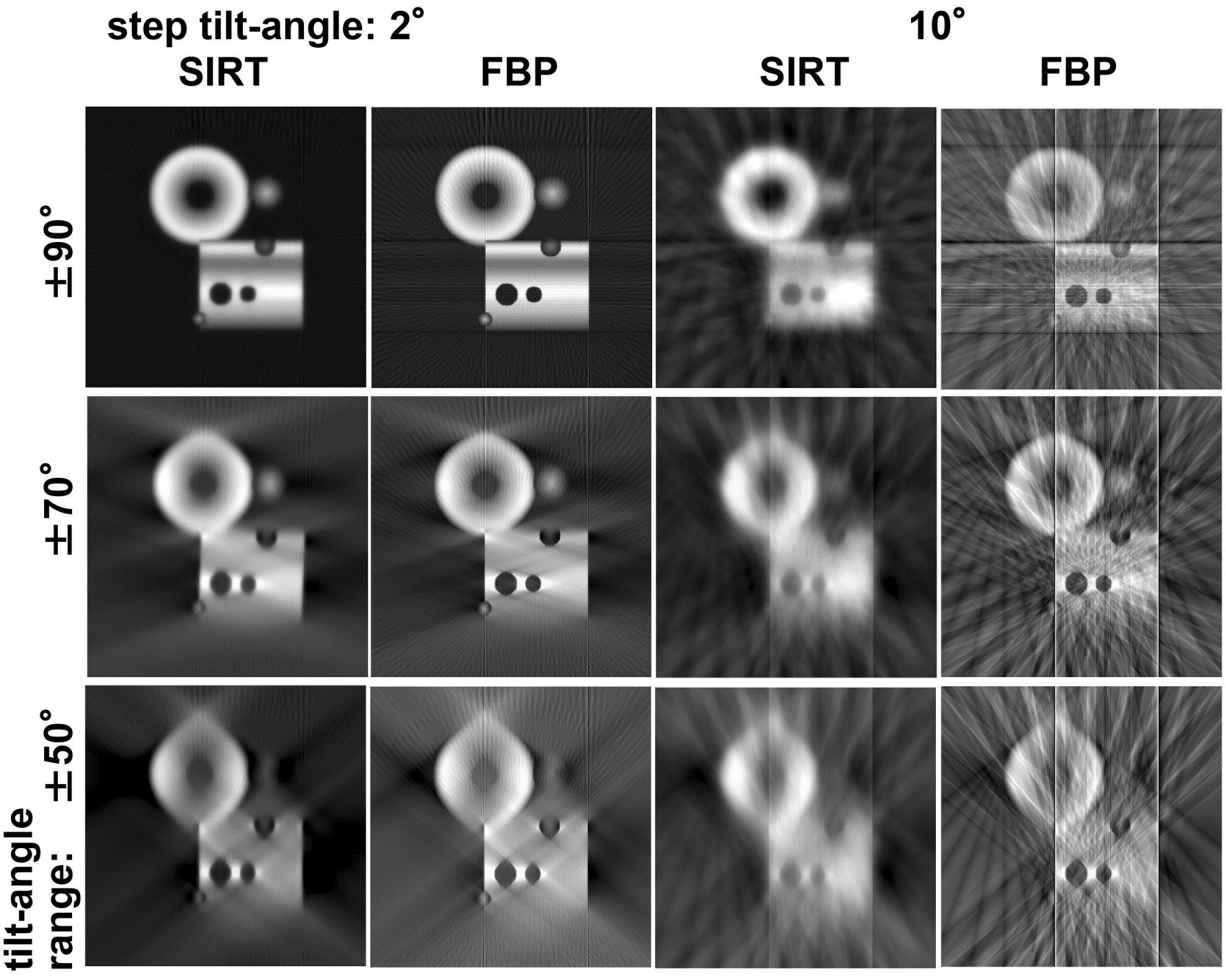
Reconstruction results of the model with SIRT and FBP for various tilt-angle conditions to compare with QURT (Fig. 8). Images are blurred and artificial background images such as streaks and clouds appear, which are attributed to the missing wedge due to the poorer conditions.

We examined various test conditions on the tilt-angle range and the step angle (Fig. 8). For comparison, Fig. 9 show the results using conventional methods of SIRT and FBP. The reconstructed results of QURT are deformed little by little from the model (Fig. 5a) due to the missing wedge. This condition deviates from the most ideal one with the ± 90° tilt angle range and a 2° step angle because the number of possible solutions (freedom of solutions) increases. However, compared to the conventional methods, QURT recovers the model relatively well.

This result can be numerically verified by calculating the average error between the model and the reconstructed results (Table 1). As a typical result, when considering the existing installation of a specimen tilting holder, which has an allowable tilt angle range of about ± 70°, our results demonstrate that the missing wedge artefact is negligible, and the structure is reconstructed almost correctly if the step angle is 5°. In addition, artificial images such as streaks and artificial background do not appear. It is noteworthy that the edge parts are clearly resolved compared with those reconstructed with the conventional methods.

**Table 1:** Comparison of average errors in model simulations between the present method (QURT) and other conventional ones.

Method		QURT	SIRT	FBP
Tilt-angle				
Range	Step			
± 90°	2°	0.00330	0.0133	0.0409
	10°	0.0116	0.0568	0.129
± 70°	2°	0.0134	0.0769	0.104
	10°	0.0189	0.0895	0.166
± 50°	2°	0.0373	0.108	0.185
	10°	0.0381	0.114	0.249

When the step angle is 10°, five plural base tilt angles of − 70°, − 50°, 0°, + 50°, and + 70° are adopted because the quality of the reconstructed images is improved compared to that using the standard three plural base tilt angles (Fig. 8). Various tests indicated that a selection of ± 50° is suitable. Considering more than five plural base tilt angles did not result in further improvement. Additionally, when the step angle is 2° or 5°, three base tilt angles is sufficient to obtain the optimum reconstruction image.

## Experiments

**Figure 10:**
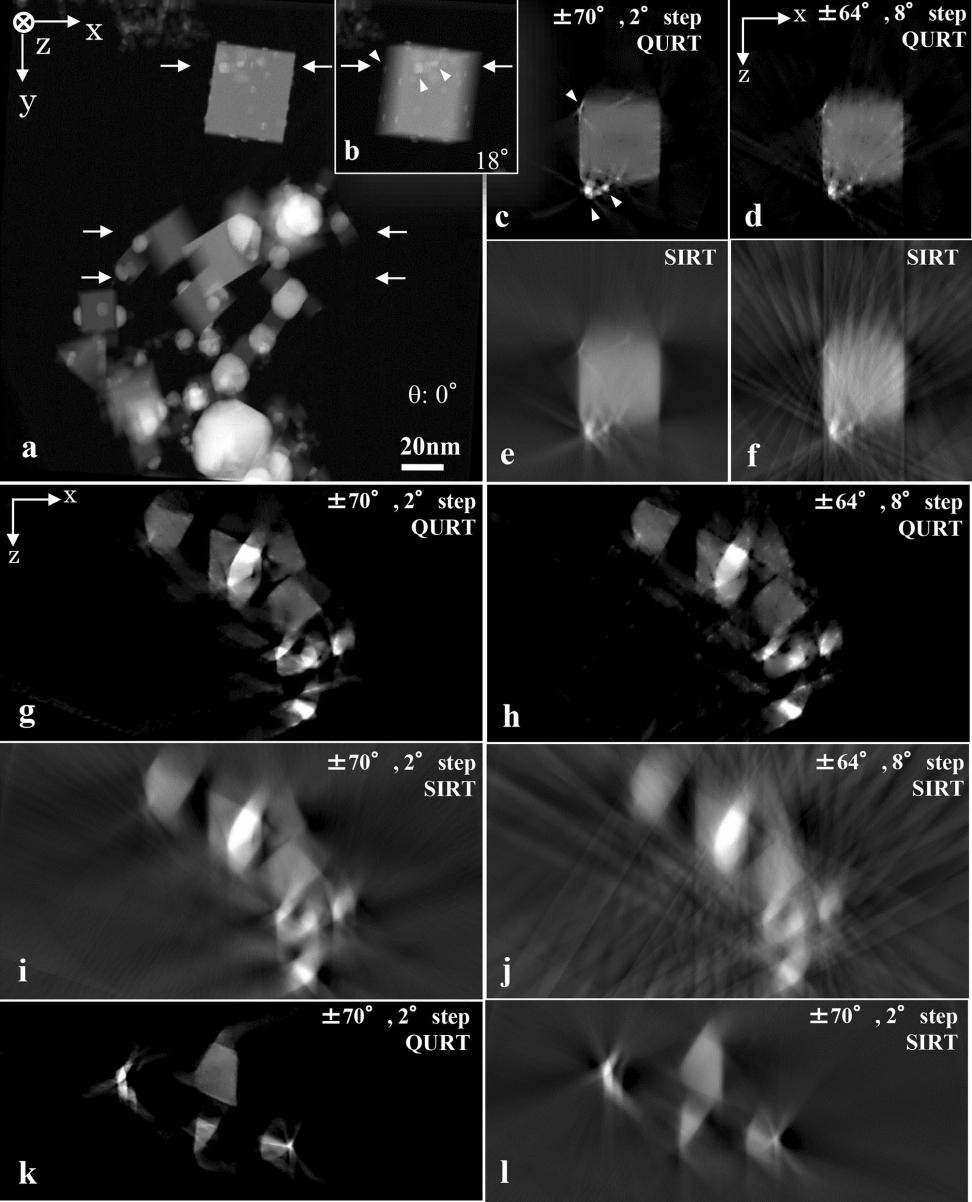
Experimental results. (**a**) HAADF STEM image of TiN-Ag nanocomposite particles taken from the 0° direction. (**b**) Small angle tilted image showing fine Ag grains spread on the surface of the TiN dice (see arrow heads). (**c** and **d**) Reconstructed results with QURT under different conditions (tilt-angle range ± 70° with 2° steps and ± 64° with 8° steps) for the cross-section indicated by the top arrows (**a**). (**e** and **f**) Results with SIRT to compare with (**c**) and (**d**), respectively. QURT clearly reconstructs the TiN dice particle with sharp edges free of the blur and artificial background due to the missing wedge in the SIRT result. In addition, Ag grains are finely reconstructed (see arrow heads) with QURT. Even if the number of projection images is reduced to a quarter, as shown in (**d**), the structure is well reconstructed compared with the very blurred SIRT result (**f**). (**g** and **h**) Other results with QURT under the same conditions of (**c**) and (**d**), respectively, for the cross-section indicated by middle arrows in (**a**). (**i** and **j**) Similar comparisons with SIRT under the same conditions of (**c**) and (**d**), respectively. (**k** and **l**) Another result with QURT for the cross-section indicated by the bottom arrows in (**a**) and comparison with SIRT, respectively. Even for the mixed many particles, QURT clearly reconstructs the morphology (**g** and **k**) without the blur and artificial background in the SIRT result (**i** and **l**). In addition, even using a quarter of the projection images, as shown in (**h**), the mixed particles structure is recovered and not affected by the missing wedge compared with the very degraded SIRT result (**j**).

QURT was applied to a practical sample of a TiN-Ag nanocomposite. Because nanocomposite particles have clear edges and geometrical forms, the sample is suitable to examine the recovery of blurred structural parts due to conventional reconstruction methods. Figure 10a shows the original STEM image (High-Angle Annular Dark Field (HAADF) image) taken from the 0° direction. The sample contains many dice-like particles. Then we acquired a series of tilt images where the range was ± 70° and the step angle was 2°.

Prior to the experimental application, the projection images of the series were adjusted. That is, the background intensity corresponding to the vacuum region, an unnecessary specimen support film image, the background of the image acquisition system, etc. were roughly subtracted from the image data. Then the image intensity was properly amplified by approximating the projection data as large integers, which were equivalent to the number of QU pieces. Although accurately subtracting the background intensity is impossible in many cases, it is necessary to get as close as possible to the theoretical constraint conditions of Eq. (3). Figure 10c shows a cross-section image of a typical section within the largest particle reconstructed using QURT and all STEM images (71 images).

QURT clearly reconstructs the base TiN dice particle. In contrast, the conventional SIRT method provides blurred sides of the contour (horizontal edges of the square shape) (Fig. 10e). QURT is unaffected by the missing wedge artefact, and artificial background images such as streaks and clouds disappear. Additionally, the fine Ag grains clearly spread on the surface of the TiN dice. The physical and material analyses are described in detail elsewhere^24^. This sharpness is a superior feature compared to conventional methods. Figure 10g,k show other cross-section images with similar features, which can be compared to the SIRT results in Fig. 10i,l, respectively.

QURT can reconstruct a structure even if the number of the tilt series images is small (Fig. 10d,h). These can be compared to the SIRT results in Fig. 10f,j, respectively. These reconstructions were calculated assuming a tilt range of ± 64° and a step angle of 8°. The SIRT blurs the structures and generates artificial background, whereas QURT well reconstructs the structures even in the worst condition. In these cases (Fig. 10d,h), five plural base tilt angles of − 64°, − 48°, 0°, + 48°, and + 64° are selected due to the improved image quality compared to that using three angles. The selection of ± 48° is determined from various tests.

## Discussion

We devised a unique method (QURT) to reconstruct a cross-section image by arranging grey-level QU pieces in a 3D image space. In the first stage (basic procedure), the predetermined number of QU pieces are placed in the negative maximum error position one-by-one in the current error map of $E_{map} \left( {x,z} \right)$ (procedure (e)). Then they are rearranged to further reduce the error amount of $E_{map} \left( {x,z} \right)$ (procedure (f)). Because the QU pieces arrangement depends on $E_{map} \left( {x,z} \right)$, this map plays an important role. However, the map is affected by the missing wedge because the initial map of $E_{map} \left( {x,z} \right)$ is calculated with the simple back projection of all data in a limited angular range.

Nevertheless, the influence on the cross-section image is minimal. In the initial stage of the basic procedure, because the error amount in the affected parts of $E_{map} \left( {x,z} \right)$ by the missing wedge is relatively small compared to those in the structural parts (Fig. 3), QU pieces are not arranged in the affected parts. Hence, the error amount in both the structural parts and the affected parts gradually becomes smaller as the QUs are not arranged in the affected parts (Fig. 3). Eventually all the QU pieces are placed, and the missing wedge artefact in the cross-section image is avoided.

This reason is essentially valid for general specimens. However, the three improvement procedures described in the ‘Reconstruction with grey-level QUs’ section are applied. Consequently, the missing wedge artefact is avoided for the general model and the experimental sample (Figs. 5, 8, 10). A remarkable result is the recovery of the Fourier spectra in the missing part (Fig. 5d). This is attributed to the restoration of the horizontal edges in the model. Moreover, the spectra in every gap between a radial line pair in the Fourier transform are recovered (Fig. 5d). In conventional reconstruction methods, Fourier spectra are limited to the radial lines based on the projection slice theorem (Fig. 5c). On the other hand, QURT is based on the arrangement of QU pieces. Consequently, the Fourier spectra do not have limitations on the radial line and the Fourier transform of the cross-section image are fully reconstructed with QURT. Therefore, QURT produces the typical ring-like diffraction pattern generated from the doughnut model even though the pattern in the missing wedge region is incomplete (Fig. 5d).

The grey-level distribution inside the general model (Fig. 5a) gradually changes from dark to bright. This is intentional to examine the applicability of the present method. The reconstruction simulation confirms that the distribution changes slightly from the model, depending on the deterioration of the tilt-angle condition. However, compared to conventional methods, QURT recovers the grey-level distribution relatively well.

This characteristic is favourable because QURT is also applicable to structures unsuitable for the sparse model adopted in CS, DART, and TVR-DART, which are based on the sparse approximation. In CS, the model is obtained by selecting the proper sparse transform such as by selecting the regularisation parameters^13^. In TVR-DART, the sparsity is given by prior knowledge about distinct material compositions within the specimen and the boundaries between different compositions in the grey-level. This algorithm starts with an initial solution for the reconstruction using either SIRT or Total Variation minimisation^11^. On the other hand, QURT does not require a specific model, approximation, or prior knowledge regarding the specimen or the expected cross-section image. It only needs the tilt series images and tilt angle data. Therefore, it is easy to use.

QURT can be categorised as a basic reconstruction method similar to FBP, WBP, and SIRT. We think that TVR-DART and CS methods are in another category of active methods, which are distinct from QURT. To improve the performance of QURT in the near future, for very poor condition projection data, it might be possible to combine QURT with an active method or to process a QURT result using an active method. In QURT, the effect of noise on projection data is mentioned below. In the algorithm, each QU size reconstruction is executed for every base tilt angle Θ which is typically three angles, but can include up to five angles, and all reconstructed results obtained from the plural Θs are averaged before the next reduced QU size reconstruction. This averaging is effective for noise reduction because each constraint condition of $n_{\Theta } \left( {x_{j}^{{\prime }} } \right)$ for Θ is affected by the noise on projections. Depending on the noise level, if the number of Θ is increased, the efficiency should be improved. Verification of this hypothesis will be reported in the near future. For further denoising, QURT may need to be combined with an active method such as the regularisation method, which is also a future research theme.

As described in the ‘Experiments’ section, the present method has realised useful results. First, it avoids both the missing wedge artefact and artificial background images. Second, it provides clear edges. These features are very useful to segment plural structural parts in post-image processing and in 3D CG demonstration of the reconstructed structure.

QURT reconstructs the structure even if the number of projection images is small, but partial incompleteness may occur. QURT is effective for applications to specimens that are susceptible to electron irradiation damage, and also effective for X-ray energy-dispersive spectroscopy (XEDS) tomography. Currently, the calculation requires a long time. In addition, for large sized projection and cross-section images, QURT requires a very large number of QU pieces. This demands a certain level of computer power. However, as computer processing technologies advance in the near future, the applicability of QURT should increase because our proposed algorithm is simple and straightforward. Although our algorithm is steady, there are alternatives to the one-by-one QU arrangement or movement method proposed here to speed up the calculation. For example, some optimisation algorithms treat the whole QU arranged distribution as one multidimensional vector. Then a cross-section image is reconstructed by obtaining the optimum solution of the vector with an algorithm such as gradient descent type one. We are planning to investigate other optimisation algorithms to improve the performance. Additionally, we will also attempt to realize adaptive change of the QU size based on the the error map $E_{map} \left( {x,z} \right)$ and an evaluation of the current error reducing rate during the iterative procedure.

## Methods

### Simulation models

A simple binary model (Fig. 3a) and a general model (Fig. 5a) were originally developed, and they are obviously affected by the missing wedge in the reconstruction using conventional methods. The binary model consisted of a doughnut and bar shape, which are assumed to be reconstructed using QU pieces with a value of 1. The general model consisted of a thick doughnut and a rectangular shape. Small holes and cones were added. The model was made with smooth grey-level distributions and steep edges. The maximum value of the model was 255, and reconstruction with QURT gave a QU piece value of 1.

### Reconstruction programs

The programs needed for QURT were custom written in our laboratory using the C++ programming language and Intel (Intel) MPI (Message Passing Interface) library (Parallel Studio XE Composer Edition for C++ Windows 2019). FBP and SIRT reconstructions were also performed using programs written in our laboratory based on the established algorithms (e.g.,^20–22^). The MPI library was used for the SIRT program to reduce the calculation time.

### Experimental data

TiN–Ag (63.98wt%TiN–36.02wt%Ag) nanocomposites were synthesised by the dc arc-plasma method, which employed a specific arc-melting chamber. The details of the chamber and the method are described elsewhere^24–26^. The nanoparticles collected from the chamber after the appropriate treatment were ultrasonically dispersed in alcohol and mounted on an amorphous carbon coated Cu grid for characterisation by transmission electron microscopy (TEM). For ET, a tilt series of projections by the STEM-HAADF method was acquired with the specimen tilt varying from − 78° to 70°. Data was recorded every 2° using a TEM operated at 300 kV (Titan 80–300, FEI, Netherlands). A STEM-HAADF detector with a specially designed high-tilt holder were attached to the TEM (E.A. Fischione Instruments, U.S.A.).

### Alignment of the tilt series

The tilt series images were accurately aligned with the method proposed in the previous paper^27^. The alignment was based on the volume cross-correlations between pairs of filtered back-projected ray volume data calculated from neighbouring projection images. The method required neither markers nor image feature points traceable through the tilt series. (See the paper for details.) A remarkable efficiency was the ability to align the series even though the step angle was larger than the normal case of a few degrees. For example, it was practically performed for steps of 8° and 10°. This is expedient when the number of projection images is small with a large step angle. Additionally, the misalignment of the tilt-axis was accurately measured, and the series was realigned with the method proposed in a previous paper^28^. This was also performed with a similar volume cross-correlation method. In practice, the misalignment of the tilt-axis was − 3.1° (counterclockwise to the vertical axis) for the present experimental tilt series.
